# Randomised controlled trial of tailored interventions to improve the management of anxiety and depressive disorders in primary care

**DOI:** 10.1186/1748-5908-6-75

**Published:** 2011-07-21

**Authors:** Henny Sinnema, Gerdien Franx, Daniëlle Volker, Cristina Majo, Berend Terluin, Michel Wensing, Anton van Balkom

**Affiliations:** 1 Netherlands Institute of Mental Health and Addiction (Trimbos-institute), Utrecht, the Netherlands; 2 The EMGO Institute for Health and Care Research (EMGO+), Amsterdam, the Netherlands; 3 Department of General Practice, VU University Medical Centre, Amsterdam, the Netherlands; 4 IQ Healthcare, Radboud University, Nijmegen, the Netherlands; 5 Department of Psychiatry, VU University Medical Centre, Amsterdam, the Netherlands

## Abstract

**Background:**

Anxiety and depressive disorders are highly prevalent disorders and are mostly treated in primary care. The management of these disorders by general practitioners is not always consistent with prevailing guidelines because of a variety of factors. Designing implementation strategies tailored to prospectively identified barriers could lead to more guideline-recommended care. Although tailoring of implementation strategies is promoted in practice, little is known about the effect on improving the quality of care for the early recognition, diagnosis, and stepped care treatment allocation in patients with anxiety or depressive disorders in general practice. This study examines whether the tailored strategy supplemented with training and feedback is more effective than providing training and feedback alone.

**Methods:**

In this cluster randomised controlled trial, a total of 22 general practices will be assigned to one of two conditions: (1) training, feedback, and tailored interventions and (2) training and feedback. The primary outcome measure is the proportion of patients who have been recognised to have anxiety and/or depressive disorder. The secondary outcome measures in patients are severity of anxiety and depressive symptoms, level of functioning, expectation towards and experience with care, quality of life, and economic costs. Measures are taken after the start of the intervention at baseline and at three- and six-month follow-ups. Secondary outcome measures in general practitioners are adherence to guideline-recommended care in care that has been delivered, the proportion of antidepressant prescriptions, and number of referrals to specialised mental healthcare facilities. Data will be gathered from the electronic medical patient records from the patients included in the study. In a process evaluation, the identification of barriers to change and the relations between prospectively identified barriers and improvement interventions selected for use will be described, as well as the factors that influence the provision of guideline-recommended care.

**Discussion:**

It is hypothesised that the adherence to guideline recommendations will be improved by designing implementation interventions that are tailored to prospectively identified barriers in the local context of general practitioners. Currently, there is insufficient evidence on the most effective and efficient approaches to tailoring, including how barriers should be identified and how interventions should be selected to address the barriers.

**Trial registration:**

NTR1912

## Background

Anxiety and depressive disorders are common mental disorders that have a negative impact on everyday functioning, cause great suffering, and incur both high healthcare costs and additional costs associated with production losses [1-3]. The lifetime prevalence of anxiety and depressive disorders in Dutch adults is about 20%, and the 12-month prevalence is 10% and 5% [4], respectively. Most adults who seek help for their anxiety or depressive disorder are treated in general practice [5,6].

In the Netherlands, clinical guidelines are available for both anxiety and depressive disorders for general practice [7-10]. Enhancing guideline adherence is expected to lead to reduction of the burden of disease and improvement of social functioning [11,12]. The management of anxiety and depressive disorders by general practitioners (GPs) is not always consistent with prevailing guidelines. Under-recognition and consequently under-treatment of anxiety and depressive disorders have been reported, where threshold disorders are more likely to be recognised than are subthreshold disorders [5,13-15]. About a quarter and a half of patients, respectively, receive optimal treatment for an anxiety disorder and a depressive disorder in primary care [16,17]. Besides under-diagnosis and under-treatment in some patients, other patients are over-treated with psychopharmacological drugs, while these are not indicated [5,18,19]. Use of effective early interventions in patients with mild problems, which are often based on cognitive behavioural techniques, is more the exception than the rule [20]. The adherence to guideline recommendations is suboptimal because of a variety of factors influencing GPs' recognition and management of anxiety and depressive disorders. These factors are related to (a) patients, such as lack of recognition of having a psychological problem, presentation of physical symptoms, absence of a perceived need for care; (b) GPs, for example, lack of knowledge and skills, attitudes, time, self-efficacy, patient-physician communication; and (c) organisation of care, such as insufficient collaboration with mental health professionals and waiting lists for specialty mental healthcare [21-26]. In addition, some recommendations in the guidelines have less support from research evidence or may be perceived as less attractive.

To improve adherence to guideline recommendations, various implementation strategies can be effective for improving professional performance in healthcare professionals [27]. Many quality-improvement interventions in anxiety and depression care target provider knowledge through education on treatment guidelines and continuous performance feedback or they contain a fixed package of multiple strategies, such as in the Quality Improvement Collaboratives [28,29]. Other interventions in anxiety and depression care target organisation of healthcare delivery, for instance, by involving mental health consultants [22,30]. The strategies show mixed and overall moderate effects on clinical management of depression and outcomes in primary care.

Our hypothesis is that adherence to guideline recommendations, and consequently patient outcomes, will be improved by designing implementation interventions that are tailored to prospectively identified barriers in the local context of GPs [23,25,31-33]. The choice of a study in tailored implementation is based on the assumption that implementation is affected by impeding local factors related to care professionals, the organisation of care, and social factors. Successful implementation is only possible when these barriers are dealt with through an implementation plan tailored to the situation [34]. Different studies have investigated the impact of tailored interventions for behaviour change in GPs, to improve the quality of care, in randomised controlled trials (RCTs) [32]. Because the tailoring methods used in these studies are heterogeneous, there is insufficient evidence on the most effective and efficient approaches to tailoring, including how barriers should be identified and how interventions should be selected to address the barriers. Therefore, we used a pragmatic and flexible approach of tailoring implementation to barriers to change.

This article describes the aims and methods of an RCT to determine the effectiveness of tailored interventions in the implementation of guideline recommendations for the early recognition, diagnosis, and stepped-care treatment allocation in patients with anxiety or depressive disorders in general practice in the Netherlands.

## Methods/design

### Objectives

The primary aim of this RCT is to determine the effectiveness of tailored interventions to improve the implementation of guideline recommendations for the early recognition, diagnosis, and stepped-care treatment allocation for anxiety and depressive disorders in general practice. Secondary aims are to describe the identification of barriers for improving professional performance, the relationship between prospectively identified barriers and improvement interventions selected for use, and the influencing factors and experiences with the strategy. The final aim is to examine the efficiency of the tailored intervention compared to usual care from a societal perspective with a time horizon of six months.

### Time frame

This study was initiated in 2009 and is planned to take 3.5 years.

### Study design

A cluster RCT with two arms has been chosen for this study. Cluster randomisation was applied at the level of the general practice organisation. The general practice organisations were allocated to the intervention or the control group. The allocation was generated by an independent statistician.

The chosen implementation strategies are

1. an educational intervention targeted at GPs, comprising of one day of training at the start and one feedback at six months (in both study arms);

2. one or more interventions tailored to prospectively identified barriers in the local context of GPs (only in the intervention arm).

### Recruitment of general practitioners

We aimed at recruiting patients and GPs in 22 general practices into our trial. Therefore, we prepared a newsletter for GPs with information about the goals of the study; the activities; and the accreditation they would receive if they followed the one-day training in guideline recommendations for the early recognition, diagnosis, and stepped-care treatment allocation of patients with anxiety or depressive disorders. Several recruitment strategies were carried out: (a) the newsletter was published at the website of the Dutch GPs association and the website of the Trimbos Institute, a centre of expertise on mental health and addiction and (b) the newsletter was sent to a sample of 500 GPs, provided by the Netherlands Institute for Health Services Research (NIVEL) and to all GPs who had a contract with a specific health insurance company that gives financial support. Subsequently, a researcher contacted all practices by phone to recommend participation. Finally, 23 general practices were included.

### Recruitment of patients

**Figure 1:**
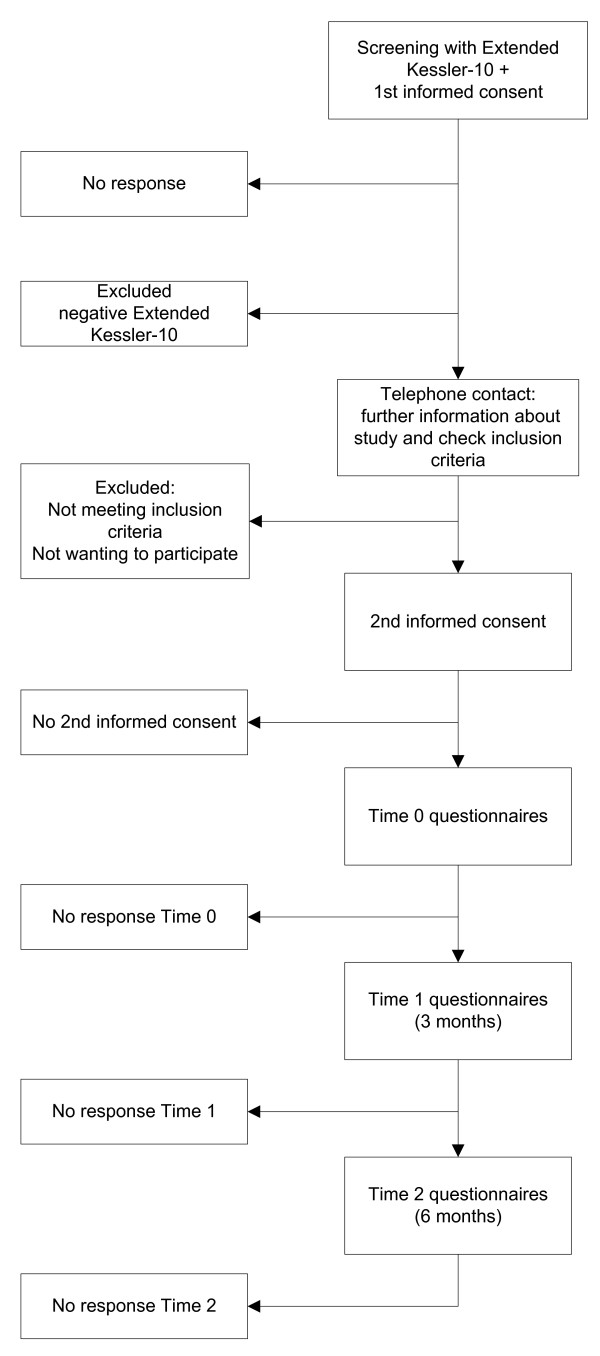
**Flowchart of participating patients**.

We aimed at including patients with symptoms that might indicate anxiety or depressive disorders. A sample of patients who visit their GP from September 2010 will receive an information letter with an invitation to participate and will be asked to fill out a short screening instrument: the extended Kessler-10 (EK-10). The Dutch EK-10 is a validated screening instrument for anxiety and depressive disorders in primary care [35]. Of those patients who return the EK-10 and give informed consent to call for the provision of further information about the study, the score on the EK-10 will be calculated. Patients are considered screen-positive if the score is 20 or higher and/or they ticked at least once a 'yes' on the added questions 11 through 16. Screen-positive patients will be called and given further information about the study. Patients who do not meet the exclusion criteria will receive a second information letter, the baseline questionnaire, and a second informed consent form. Patients will be given the option to complete the questionnaire in writing or digitally. Inclusion in the study will be definite if the patient returns the baseline questionnaire and gives informed consent for participation in the study. GPs are not informed about the inclusion of their patient. Figure 1 shows a flowchart of participating patients.

### Patient inclusion and exclusion criteria

Inclusion criteria are an age of 18 years and older, a score on the Dutch version of the EK-10 of 20 or higher, and/or at least one yes on the added questions 11 through 16. Exclusion criteria are an age under 18 years, suicidal ideations, dementia or other severe cognitive disorders, psychotic disorder, bipolar disorder, dependence on alcohol or drugs, unstable severe medical condition as diagnosed by their GP, insufficient knowledge of the Dutch language to fill out the questionnaires, or having received psychological treatment in the six months before the start of the study or recognised with anxiety or depressive symptoms by their GPs in this period.

### Sample size

The primary outcome measure for the evaluation of the effectiveness of tailored interventions is the recognition of anxiety or depressive disorders by GPs in patients with symptoms that might indicate these conditions. The rate of recognition was reported at about 45% [14,36]. Studies showed that interventions focused on professionals' adherence to guidelines can increase adherence by 10% [27]. With tailored interventions, we suppose the recognition can improve by 15%. To get an accurate estimate (alpha = 0.05; power = 0.80) of a 15% difference (45% vs. 60%) in recognition between both groups, assuming that 5% of participants will drop out (loss to follow-up will be minimal because we will perform a retrospective medical record search to get insight in rate of recognition) and considering an intracluster correlation of 0.01 [37], 396 patients in 22 practices have to be included.

### Intervention group

#### Tailored strategies

GPs from the general practices randomised to the intervention group will receive interventions that are tailored to prospectively identified barriers in their local context over the course of one year. Methods for tailoring implementation interventions to local barriers vary widely and are often poorly documented [31]. To get insight in the experienced barriers in the early recognition, appropriate diagnosis, stepped-care treatment allocation, and providing of information on the diagnosis and stepped-care treatment options for anxiety and depressive disorders, a semistructured face-to-face interview was carried out with each of the participating GPs by a trained interviewer. For this interview, we developed a checklist based on the main types of barriers to adherence to evidence-based guidelines on anxiety and depressive disorders by GPs [25,28,38]. Based on these main types of barriers, we developed interventions that could solve the barriers. Each interview was documented in a report. Based on this report, experts suggest interventions that may resolve the barriers. These interventions are fed back by telephone to the GP by the same interviewer.

The interviewer calls the GP once every two months to map the implementation process and links this back to the experts. Again, experts suggest interventions or give advice to the interviewer for the next contact with the GP. With a continuous feedback loop between the experts, the interviewer, and the GP, we optimise the tailoring process. All contacts between the experts, the GP visitor, and the GP are reported.

#### Training

GPs in both conditions received one day of training by experts in (a) the early recognition of high-risk patients with the Four-Dimensional Symptom Questionnaire (4DSQ), (b) appropriate diagnosis, (c) stepped-care treatment allocation, and (d) the providing of information to patients with anxiety and depressive disorders. The 4DSQ is a self-rating questionnaire measuring four dimensions of common psychopathology: distress, depression, anxiety, and somatization. The 4DSQ was developed in general practice. The principal aim of the 4DSQ is to distinguish between stress-related syndromes (denoted as 'stress', 'burnout', 'nervous breakdown') and psychiatric disorders (*i.e*., depression and anxiety disorders) [39]. The 4DSQ can be used in recognising high-risk patients for anxiety or depressive disorders and is recommended by the multidisciplinary guidelines on anxiety and depressive disorders. Criteria for high-risk patients are described in the clinical guidelines for anxiety and depressive disorders for general practice. In a former quality-improvement project, GPs showed positive experiences with the use of the 4DSQ in detecting anxiety disorders. The 4DSQ offers GPs a means to start talking with patients with unexplained somatic symptoms about possible psychological or psychiatric disorders.

Adequate diagnosis is based on the recommendations of clinical guidelines for general practice. Stepped-care treatment allocation is based on the multidisciplinary guidelines. According to the stepped-care model, patients with a noncomplex anxiety disorder or a nonsevere depressive disorder have to receive as a first step brief interventions, such as guided self-help or brief therapy. Patients with a complex anxiety disorder or a severe depressive disorder have to receive effective psychotherapeutic interventions, an antidepressant, or a combination of both. Determination of the complexity of an anxiety disorder is based on at least one of the following criteria: serious social/functional dysfunction, comorbidity (patient has another anxiety disorder or depression), obsessive compulsive disorder or posttraumatic stress disorder, no response after a minimum of six weeks and maximum 18 weeks, or no remission after a first-step brief intervention. Determination of the severity of a depressive disorder is based on at least one of the following criteria: high level of distress, serious social/functional dysfunction, minimum eight symptoms of the depressive disorder according to the fourth edition of the *Diagnostic and Statistical Manual of Mental Disorders *(DSM-IV) [40], psychotic features, suicidal ideation.

#### Feedback

GPs in both conditions were asked to fill out a consultation registration form for each patient who completed the 4DSQ. On this form, GPs need to register the score on the 4DSQ, the diagnosis, the indicated treatment, and if they informed the patient about the diagnosis and stepped-care treatment options. GPs received individual feedback on the number of registered 4DSQs, appropriate diagnosis, stepped-care treatment allocation, and information on the diagnosis and stepped-care treatment options in a report after six months, based on the consultation registration forms.

#### Control group

GPs from the general practices randomised to the control group only received training and feedback (see intervention group).

### Outcome measures

#### Primary outcome

The primary outcome for both conditions is the proportion of patients who have been appropriately recognised to have anxiety and/or depressive disorder. This proportion is calculated by dividing the amount of patients recognised by the GP by the total amount of patients included in the study. Recognition is measured by the registration of (a) anxiety or depressive complaints; (b) psychological complaints (anxiety, worrying, sorrow/grief, stress, feeling down and sleeping disorder, unexplainable somatic complaints); (c) the International Classification of Primary Care-1 (ICPC-1) codes [41] for anxiety and/or depression and/or related psychological problems, the same ICPC codes that were used in previous work by Smolders [17]; and (d) the 4DSQ score.

### Secondary outcomes

#### In patients

The secondary outcome measures in patients are severity of anxiety and depressive symptoms measured with the 4DSQ; level of functioning, measured with the World Health Organization's Disability Assessment Scale II [42]; expectation towards and experience with care, measured with the QUality Of care Through the Eyes of the patient (QUOTE) [43]; care utilisation, illness, and work, measured with the Trimbos/institute Medical Technology Assessment questionnaire for Costs associated with Psychiatric Illness (TiC-P) [44]; and quality of life, measured with the EuroQol (EQ-5D) [45]. Measurement will take place every three months: at baseline (T0) and at three (T1) and six months (T2) after inclusion.

#### In general practice

The secondary outcome measures for both conditions are proportion of patients for whom tricyclic antidepressants (TCA's) or selective serotonin reuptake inhibitors were prescribed and number of referrals to specialised mental healthcare. We gather data to calculate the secondary outcomes by performing a retrospective patient medical record search, after the last patient's follow-up measure.

#### Process evaluation

In a process evaluation, we describe the identification of barriers to change and the relationships between prospectively identified barriers and improvement interventions selected for use in the intervention group. We evaluate the experiences of GPs in the intervention group, the GP visitors and the experts with the tailoring process, the implemented changes in practice, and the factors influencing the tailoring process. To measure the experiences, semistructured interviews are conducted, and reports of the interviews are made.

To get an insight into the factors that influence the provision of guideline-recommended care in both groups, all GPs are asked to fill out an individual self-administered questionnaire about the general practice and GP characteristics. The practice characteristics include practice type, number of GPs in the practice, collaboration with other healthcare professionals working in the practice, and size of practice population. The GP characteristics include demographic data, interest and attitudes towards depressive and anxiety disorders, and questions to assess barriers to healthcare provision to patients with depressive or anxiety disorders and, for implementation of the depression and anxiety guidelines, collaboration with professionals and institutions specialised in mental healthcare and GPs' levels of burnout. This questionnaire is developed and used in the Netherlands Study of Depression and Anxiety, an eight-year longitudinal cohort study designed to be representative of persons with depressive and anxiety disorders in different healthcare settings and in different stages of the disorders [24,46]. The questionnaire is filled out twice: before the start of the tailoring process and when the tailoring process is finished.

#### Economic evaluation

An economic evaluation will be conducted to estimate the cost effectiveness of the tailored intervention from a societal perspective. The between-group difference in costs will be related to the difference in benefits in terms of health-related utilities. This economic evaluation uses the EQ-5D. The cost-utility analysis measures health in quality-adjusted life years (QALYs), derived using the EQ-5D questionnaire [47]. The EQ-5D characterises five health dimensions (mobility, self-care, usual activities, pain, and anxiety/depression), each rated using three levels (no problems, moderate, and severe problems). Responses will be transformed into a health utility score that ranges between 0 = dead and 1 = full health. Health utilities will then be used to calculate the QALYs. This gives a comparison of how many QALYs individuals in each group gained on average as a result of the tailored intervention. Results can statistically be compared to see if there are any differences. The final step of the cost-utility analysis will be to compare the cost of the QALY gains in each group.

The costs of the implementation strategy used will be studied for each practice. These costs are (a) the costs per hour of the GP visitor and experts for the activities during the tailoring process and the one-day training and feedback, (b) material costs for the one-day training, and (c) the costs per hour of the GP related to the implemented interventions. In addition, the difference in healthcare costs related to the diagnostic process will be included, including number of consultations, diagnostic tests, and referrals with diagnostic aims. Healthcare costs will be measured by the TiC-P. The costs will be estimated in line with the Dutch guidelines for cost calculations in healthcare [48].

Apart from costs of the tailored strategy, healthcare costs, and costs of production losses, indirect costs will be measured for both groups related to the severity of anxiety and depressive symptoms, level of functioning, experience with care, and quality of life.

### Statistical analyses

The adequacy of randomisation is assessed comparing characteristics of the general practice and GPs that might influence the outcomes (see process evaluation). Recognition of anxiety or depressive disorders in eligible patients in the study period is compared between the intervention and control groups, taking into account clustering of data in a multilevel regression model. We will also calculate and compare the percentages of patients who have been appropriately recognised and diagnosed, prescribed antidepressants, and referred and the number of consultations, determined by the registration in patients' medical records.

Descriptive statistics will be used to outline the characteristics of practices and GPs. Finally, bivariate and multivariate multilevel regression analyses will be performed to identify factors associated with better adherence to specific guideline recommendations. All analyses will be performed on an intention-to-treat basis. Possible confounding characteristics (*e.g*., age, gender) will be included in the statistical models.

In addition, we will describe the barriers for change that were identified and the relationships between prospectively identified barriers and improvement interventions selected for use, based on an analysis of the records from the contacts between the GP and the interviewer. Data about the experiences of participants in the tailoring process, the implemented changes in practice, and their ideas about influencing factors will be structured, interpreted, and described in a qualitative way.

Direct and indirect costs of the interventions will be reported. The results of the cost will be presented as mean values with standard errors. Cost effectiveness will be presented in incremental cost-effectiveness ratios. The uncertainty will be assessed using bootstrapping, and acceptability curves will be presented [49]. A principled method for dealing with missing data will be applied to the economic evaluation [50].

### Ethical principles

The study protocol has been approved by the Medical Ethical Committee of the Institutions for Mental Health (METiGG; Utrecht, the Netherlands) in 2009.

## Discussion

Early recognition, diagnosis, and stepped-care treatment allocation in patients with anxiety or depressive disorders in general practice is dependent on a variety of factors influencing GP performance. The study gives information about the relevant barriers for improvement and whether they differ between GPs. Designing implementation interventions that are tailored to prospectively identified barriers for improvement in the local context of GPs could lead to more guideline-recommended care. Different studies have investigated the impact of tailored interventions for behaviour change in GPs, to improve the quality of care, in an RCT, but little is known about what methods and models of tailoring are effective and efficient [31,32]. The aim of this RCT is to determine the effectiveness of tailored interventions to improve the implementation of guideline recommendations for the early recognition, diagnosis, and stepped-care treatment allocation for anxiety and depressive disorders in primary care and describe the methods used in the process of tailoring. Because the performance of GPs during the tailoring process may be influenced by policy developments, personal attention, or even external financial incentives, the relationships between prospectively identified barriers and improvement interventions selected for use will be described, as well as the factors that influence the provision of guideline-recommended care.

## Competing interests

MW is an Associate Editor of Implementation Science. All decisions on this manuscript were made by another Editor. The authors declare that they have no other competing interests.

## Authors' contributions

AVB contributed to the design of the study and coauthored the article. MW contributed to the design of the study and coauthored the article. BT contributed to the design of the study, participated in the training of GPs, and coauthored this article. CM contributed to the design of the economic evaluation and coauthored the article. DV coauthored the article. GF contributed to the design of the study and coauthored the article. HS contributed to the design of the study and wrote this article.

All authors have read and approved the final manuscript.
